# Investigating group A *Streptococcus* antibiotic tolerance in necrotizing fasciitis

**DOI:** 10.1128/msphere.00634-24

**Published:** 2024-08-27

**Authors:** Nadia Keller, Mathilde Boumasmoud, Federica Andreoni, Andrea Tarnutzer, Manuela von Matt, Thomas C. Scheier, Jana Epprecht, David Weller, Alejandro Gómez-Mejia, Markus Huemer, Donata von Reibnitz, Duveken B. Y. Fontein, Ewerton Marques-Maggio, Reto A. Schuepbach, Srikanth Mairpady-Shambat, Silvio D. Brugger, Annelies S. Zinkernagel

**Affiliations:** 1 Department of Infectious Diseases and Hospital Epidemiology, University Hospital Zurich, University of Zurich, Zurich, Switzerland; 2 Department of Plastic Surgery and Hand Surgery, University Hospital Zurich, University of Zurich, Zurich, Switzerland; 3 Division of Clinical Pathology, University Hospital Zurich, University of Zurich, Zurich, Switzerland; 4 Institute of Intensive Care Medicine, University Hospital Zurich, University of Zurich, Zurich, Switzerland; University of Nebraska Medical Center College of Medicine, Omaha, Nebraska, USA

**Keywords:** group A *Streptococcus*, necrotizing fasciitis, tolerance, persisters, dormancy

## Abstract

Group A *Streptococcus* (GAS) necrotizing fasciitis (NF) is a difficult-to-treat bacterial infection associated with high morbidity and mortality despite extensive surgery and targeted antibiotic treatment. Difficult-to-treat infections are often characterized by the presence of bacteria surviving prolonged antibiotic exposure without displaying genetic resistance, referred to as persisters. In the present study, we investigated the presence of GAS persisters in tissue freshly debrided from patients as well as in an *in vivo* mouse model of NF and examined the phenomenon of antibiotic tolerance. Time-lapse imaging of GAS plated directly upon isolation from NF debrided tissue and an antibiotic challenge-based persisters assay were used to assess the presence of persisters. We show for the first time that GAS recovered directly from freshly debrided NF tissue is characterized by heterogeneous and overall delayed colony appearance time, suggesting the presence of persisters. Acidic pH or nutrient stress exposure, mimicking the NF-like environment *in vitro*, led to a similar phenotypic heterogeneity and resulted in enhanced survival upon antibiotic challenge, confirming the presence of GAS persisters. GAS persisters might contribute to NF treatment failure, despite extensive surgery and adequate antibiotic treatment.

**IMPORTANCE:**

Difficult-to-treat and recurrent infections are a global problem burdening society and the health care system alike. Unraveling the mechanisms by which bacteria can survive antibiotic treatment without developing genetic resistance is of utmost importance to lay the foundation for new, effective therapeutic approaches. For the first time, we describe the phenomenon of antibiotic tolerance in group A *Streptococcus* (GAS) isolated from necrotizing fasciitis (NF) patients. Dormant, non-replicating cells (persisters) are tolerant to antibiotics and their occurrence *in vivo* is reported in an increasing number of bacterial species. Tailored treatment options, including the use of persisters-targeting drugs, need to be developed to specifically target dormant bacteria causing difficult-to-treat and recurrent infections.

## INTRODUCTION

Necrotizing fasciitis (NF) caused by the human pathogen Group A *Streptococcus* (GAS, aka *Streptococcus pyogenes*) remains a devastating disease, associated with high morbidity and mortality despite the use of effective antibiotics and extensive surgery (1). Bacteria employ several strategies to escape killing, including the evolution of antibiotic resistance, intracellular localization, and biofilm formation, granting protection from antibiotic exposure and the immune system (2). Antibiotic tolerance describes the ability of a fully antibiotic-susceptible bacterial population to survive antibiotic exposure for a prolonged time (3). Individual surviving bacteria, called persisters, are characterized by an altered metabolism allowing them to withstand adverse conditions and cause relapsing infections after antibiotics removal. Antibiotic persistence has been extensively studied in Gram-negative as well as Gram-positive bacteria (4–6). Upon plating on nutrient-rich agar, persisters are characterized by a delay in growth resumption as compared to actively growing bacteria. Therefore, monitoring colony appearance time is a way to detect their presence in clinical samples (5, 7–9).

The presence of persisters in invasive GAS infections has not been investigated yet. We, therefore, monitored colony growth of GAS immediately upon isolation from NF patients and mice as well as quantified survival to *in vitro* antibiotic challenge upon pre-exposure to acidic pH stress and/or nutrient starvation. Here, we describe for the first time the phenomenon of antibiotic tolerance in GAS-NF, adding another possible explanation for antibiotic treatment failure and why surgical debridement remains essential in treating GAS-NF.

## MATERIALS AND METHODS

### Patient information and sample processing

Six patients presenting with GAS-NF at the University Hospital Zurich and from whom tissue samples were obtained immediately after surgery were enrolled in this study between June 2017 and December 2023. Patient parameters, including clinical isolate information and treatment regimens, are depicted in Table 1.

**TABLE 1:** Patients and strain information^*a*^

Patient ID Sex (F/M) Age (years)	Isolate ID *emm*-type St-type	Material	ICU stay (days)	Hospital stay (days)	Elapsed time between the appearance of first symptoms and medical intervention **Complications**	Outcome	Treatment regimen			
							Drug	Days after surgery	Treatment duration (days)	IVIG
1 F 66	CI1316 *emm*-1 St-28	Tissue	4	18	1 day Septic shock Bacteremia Acute kidney failure Mechanical ventilation (1 day) Vasopressors (1 day)	Survival	CLI	0	5	Yes
							CTR	0	15	
							CIP DOX	NA NA	NA NA	
2 M 36	CI1453 *emm*-1 St-28	Axillary lymph-node	2	9	1 day Mild, type I respiratory failure (oxygen therapy)	Survival	CTR	0	9	Yes
							CLI	0	17	
							CTR	7	1	
3 M 50	CI2261 *emm*-1 New St-type	Tissue	21	61	8 days Septic shock Bacteremia Acute or chronic kidney failure necessitating dialysis (2 days) Liver failure Mechanical ventilation (1 day) Vasopressors (1.5 days)	Survival	CLI PIT CTR MER CLI PIT MER	0 0 6 11 11 20 24	2 6 5 6 6 4 1	Yes
4 F 63	CI8223 *emm*-1 St-28	Tissue	4	23	4 days Sepsis Bacteremia Takotsubo-cardiomyopathy and atrial fibrillation with heart failure Vasopressors (1 day)	Survival	CLI CTR AMC	0 0 3	10 4 18	No
5 F 76	CI8249 *emm*-1 St-28	Tissue	3	22	3 days Bacteremia Acute kidney failure Postoperative hematoma	Survival	AMC CTR CLI	0 0 0	2 13 24	Yes
6 M 25	CI9419 *emm*-141 (closest hit) St-371	Tissue	2	NA	4 days Sepsis Bacteremia Mechanical ventilation (1 day) Vasopressors (2 days)	Survival	PIT CTR CLI	0 0 0	1 NA NA	Yes

^*a*^ If antibiotic therapy was started before surgery, the symbol “0” is depicted in the column “days after surgery.” Abbreviations: AMC, amoxicillin/clavulanic acid; CIP, ciprofloxacin; CLI, clindamycin; CTR, ceftriaxone; CUR, cefuroxime; DOX, doxycycline; MER, meropenem; PIT, piperacillin/tazobactam; NA, not available; IVIG, intravenous immunoglobulin. CI1316, CI8223 and CI9419 presented a mutation of the CovR/S regulatory system.

Patient tissue samples were processed immediately after surgery for isolation of bacteria and monitoring of colony appearance time. Tissue samples were cut into small pieces (<1 mm) and PBS was added at a 50% wt to volume ratio. The samples were homogenized using a tissue lyser (Qiagen), the bacterial fraction collected, washed two times in PBS, and finally resuspended in 500 µL of deionized sterile water, to favor lysis of residual eukaryotic cells. Ten-fold serial dilutions were spread-plated on 5% sheep blood Columbia agar plates (COS, BioMérieux) and incubated at 37°C for time-lapse imaging (5, 10). All colonies that grew on the plates used for time-lapse were pooled and stored in THY+ 40% glycerol at −80°C.

Patient tissue was processed as previously described for histological evaluation (11). In brief, patient tissue was fixed in buffered formalin (4%) and subsequently embedded in paraffin, cut into sections of 2 µm, and stained with hematoxylin and eosin or Brown-Brenn. A board-certified pathologist carried out the histological evaluation of the slides.

### Mouse experiments

C57BL/6 WT (Javier Labs, Le Genest-Saint-Isle France) mice were handled in strict accordance with the Swiss Federal Veterinary Office guidelines, and the protocols ZH251/14 and ZH050/18 were approved by the Institutional Animal Care and Use Committee of the University of Zurich and the Cantonal Veterinary Office in Zurich (Veterinäramt, Gesundheitsdirektion Kanton Zürich). *In vivo* infections were performed as previously described (11). Seven to nine weeks-old female mice were injected subcutaneously in each leg with 3 × 10^7^ CFUs of log phase GAS M1T1 5448 (GAS WT) (12) mixed with cytodex-beads (Sigma-Aldrich). Three days post-infection mice were sacrificed and skin and lymph nodes were removed for further processing and bacterial isolation, as described above for patient samples. Bacterial suspensions were spread-plated on COS plates and incubated at 37°C for subsequent time-lapse analysis.

### Colony imaging and appearance time definition

Colony growth was monitored with an automated imaging system, acquiring pictures of the blood agar plates at 10-minute intervals (7). Downstream analysis was performed with ColTapp (7). Colony appearance time, defined as the time to reach a radius of 200 µm, was derived from the colony radial growth curves.

### Bacterial growth conditions

GAS M1T1 5448 (GAS WT), its animal passaged version (GAS AP) carrying a mutation in the *covS* gene, leading to upregulation of virulence factors expression (13), and the six clinical isolates were cultivated in Todd Hewitt Broth (THB; BD) supplemented with 2% yeast extract (THY; Oxoid) and grown at 37°C in a static incubator. CI1316, CI1453, CI2261, CI8223, CI8249, and CI9419 were isolated at the University Hospital Zurich from NF patient material (Table 1). For acidic pH stress, bacteria were grown overnight (O/N) on COS plates, resuspended in pH6 medium [60% THY + 30% H_2_O + 10% pH 4.6 buffer (46.8% Na_2_HPO_4_ 0.2 M + 53.2% citric acid 0.1 M)] to an optical density at 600 nm (OD_600_) of either 0.2 for GAS WT, CI1453, and CI2261 or 2.0 for GAS AP and CI1326 and incubated at 37°C for 24 hours. For neutral pH exposure, bacteria were grown O/N on COS plates, resuspended in pH7.4 medium [60% THY + 30% pH 7.5 buffer (90.85% Na_2_HPO_4_ 0.2 M + 9.15% citric acid 0.1 M)] to an OD_600_ of 0.2 and incubated at 37°C for 24 hours. Serial dilutions of all cultures were subsequently plated and incubated at 37°C for colony enumeration (THY agar) and monitoring of colony appearance time (COS plates). Survival was calculated based on the initial inoculum.

### Antibiotic susceptibility evaluation

The minimal inhibitory concentration (MIC) of ceftriaxone was determined with the broth microdilution assay in THY medium (14). The MIC was 0.0625 ug/mL for all strains.

### Persisters assay and time-kill curves

Persisters assays were carried out on exponential growth-phase bacteria, stationary growth-phase bacteria, or bacteria exposed to acidic pH, the latter described above. Exponential growth-phase bacteria were obtained by diluting O/N cultures 1:100 in fresh THY and allowing regrowth to an OD_600_ of 0.4. Stationary growth-phase bacteria, subject to nutrient depletion, were sampled directly from a COS plate or a THY liquid culture after O/N growth at 37°C.

A total of 5 × 10^7^ colony forming units (CFUs)/mL were challenged with 40× MIC ceftriaxone in THY medium and incubated at 37°C for 24 hours. The fraction of antibiotic-tolerant cells was assessed by monitoring bacterial survival at 24 hours. Time-kill dynamics were captured by repeating the same procedure on stationary phase bacteria grown O/N in THY and monitoring bacterial survival at 2, 4, 6, 8, and 24 hours. Bacterial pellets were washed three times in 1 mL PBS to avoid carryover of antibiotics. Ten-fold serial dilutions of the bacterial suspensions were subsequently plated on THY agar plates. Survival was calculated as a percentage of the initial inoculum.

### Phylogenetic analysis and genotyping

The six isolates were whole-genome sequenced as previously described (15). All Illumina paired-end reads are available through the European Nucleotide Archive project PRJEB52606. Briefly, *de novo* assemblies were generated with Unicycler (16) and annotated with Prokka (17). A pangenome was constructed with Roary (18) using the command -e –mafft to obtain a core genome alignment, which was used as input to fasttree (19) to build a maximum likelihood tree (Fig. S1). Sequence type determination and typing of the *emm* and *covS* genes were performed *in silico* using Blastn 2.9.0 as previously described (20).

### Statistics

To determine the effect of clinical isolate identity and condition on median colony appearance time, a two-way analysis of variance (ANOVA) was performed in R 4.1.3. Subsequently, specific pairwise comparisons were computed by estimating marginal post hoc tests (multivariate t-distribution based *P*-value correction, emmeans package). To analyze the statistical significance of survival upon antibiotic challenge, the ratio paired *t*-test was used (GraphPad).

## RESULTS

### Patients and clinical isolates

**Fig 1:**
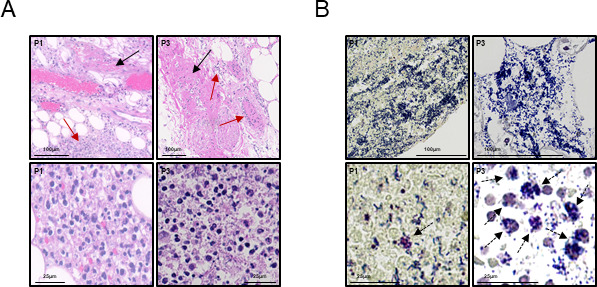
Histology. (**A**) Hematoxylin-eosin stain. Upper panels: the black arrows indicate fibrin clots; the red arrows indicate inflammatory infiltrates. Lower panels: inflammatory infiltrates mainly constituted by neutrophils. (**B**) Brown-Brenn stain. Upper panels: dense biofilm-like bacterial aggregates present in the tissue. Lower panels: the dashed arrows indicate intracellularly located GAS. P1, patient 1; P3, patient 3.

Six patients with GAS-NF were enrolled in this study and the corresponding GAS isolates were retrieved for analysis (Table 1). Histological analysis showed inflammation and infiltration of immune cells into the tissue (Fig. 1A), fibrin clots (Fig. 1A-top), and bacteria in clusters (Fig. 1B-top) as well as engulfed within immune cells (Fig. 1B-bottom). Most isolates were of *emm*-type 1 and sequence-type (ST)-28, a prevalent serotype in invasive infections (21), and genetically closely related. CI9419 was *emm*-type 141 (closest hit, with >92% identity) and ST-371 and genetically distantly related to the *emm*-1 isolates clade (Fig. S1). CI1316, CI8223, and CI9419 harbored a non-synonymous mutation in the *covS* gene, resulting in a truncated CovS protein (Fig. S2).

### Delayed colony appearance time in GAS freshly isolated from patients and mice

**Fig 2:**
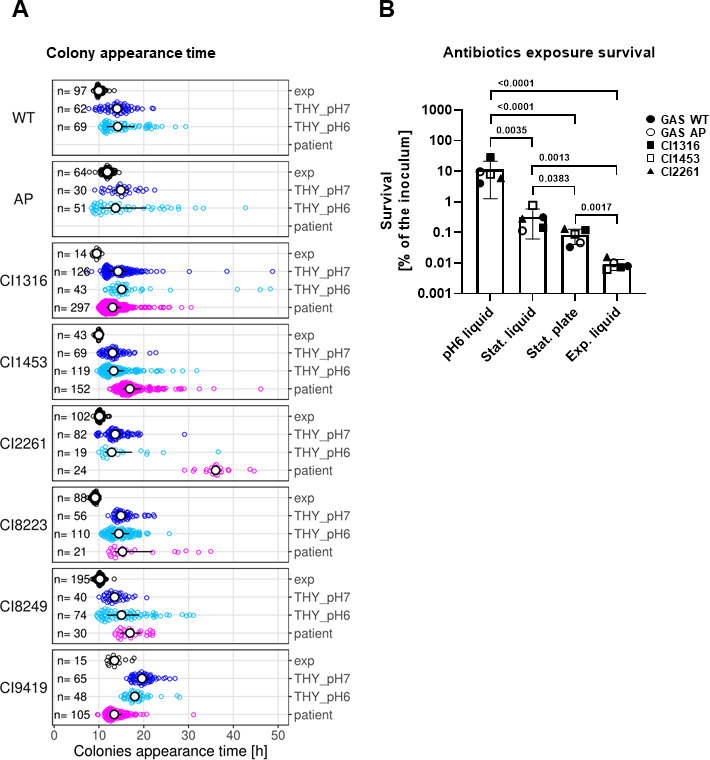
Clinical isolates lag-time and antibiotic tolerance. (**A**) Colony appearance time distributions of GAS M1T1 5448 WT (WT), its isogenic covS mutant GAS AP (AP), and the six clinical isolates derived from either exponential growth-phase (exp) or stationary cultures exposed to neutral pH (THY_pH7) or acidic pH (THY_pH6) are shown. Additionally, for the six clinical isolates, the distribution obtained directly upon sampling from the patient is shown (patient). Empty dots represent individual colonies, and the number of colonies on each plate is shown on the left (n). The interquartile range and median of each distribution are depicted with a black line and a white dot, respectively. A two-way ANOVA revealed that median appearance-time did not vary among clinical isolates (F_7,19_ = 0.65, *P* = 0.71), but varied among conditions (F_3,19_ = 4.1, *P* = 0.02). Estimated marginal means post hoc *tests* were performed to compare the median appearance-time averaged across isolates of exponential cultures against other conditions (exp vs THY_pH7, *P*-value = 0.16; exp vs THY_pH6, *P*-value = 0.18; exp vs patient *P*-value = 0.090; *P*-value corrections based on multivariate t-distribution, emmeans package). (**B**) Antibiotic survival of the various strains after exposure to acidic pH stress for 24 hours (pH6 liquid, THY) and sampled either from stationary growth-phase cultures in liquid (Stat. liquid, THY) or solid medium (Stat. plate, COS plate), or from exponential growth-phase cultures (Exp. liquid, THY). Each data point represents the average of at least three biological replicates. Statistical analysis was carried out using the ratio-paired *t*-test. *P*-values are indicated on the graph.

GAS was plated immediately upon isolation from patient tissue obtained during surgery (all clinical isolates) or debrided mouse tissue after infection (GAS WT) and colony appearance time was monitored (Fig. 2A; Fig. S3). Subsequently, the bacteria isolated were cultured and plated either upon exponential growth-phase or after 24 hours exposure to acidic or neutral pH and nutrient starvation (Fig. 2A). Despite the genetic variation, including mutations in the *covS* gene in half of the strains (GAS AP, CI1316, CI8223, CI9419), median colony appearance time was unaffected by clinical isolate identity (F_7,19_ = 0.65, *P* = 0.71). However, the median colony appearance time significantly varied among conditions (F_3,19_ = 4.1, *P* = 0.02). Exponential growth-phase bacteria formed colonies detectable after 10 hours of incubation while bacteria recovered directly from patient and mouse samples or exposed to acidic or neutral pH for 24 hours displayed delayed colony appearance times (Fig. 2A; Fig. S3).

### Acidic pH and nutrient starvation enhance antibiotic tolerance *in vitro*

To verify whether bacteria subject to *in vitro* stress and displaying delays in growth resumption were characterized by increased antibiotic tolerance, we estimated their survival rate upon 24 hours challenge with 40× MIC of ceftriaxone and compared it with the baseline survival rate of exponentially growing bacteria (Fig. 2B). Bacteria from stationary growth-phase, mimicking nutrient starvation, sampled either from O/N growth in liquid culture or on COS plates, survived significantly better as compared to their exponential growth-phase counterparts (~0.1% and ~0.3% survival, respectively, vs ~0.01% survival) (Fig. 2B). Acidic pH pre-exposure triggered a significantly increased survival (~10% survival), indicating the presence of a higher number of persisters in the overall bacterial population (Fig. 2B). Time-kill curves, carried out during exposure to 40× MIC of ceftriaxone, showed a sharp decline in bacterial viability during the first 2–4 hours of incubation (Fig. S4). The killing rate then declined to reach a plateau at around 8 hours of growth, indicating biphasic killing.

## DISCUSSION

We show for the first time delays in growth resumption in GAS isolated directly from tissue immediately after surgical debridement of patients suffering from NF as well as in mice indicating the presence of a dormant sub-population of persisters. Recreating acidic pH stress and/or nutrient starvation *in vitro* also led to increased heterogeneity in colony appearance time, confirming that these stressors can trigger persisters formation in GAS. This was reflected by increased survival rates when the stressors were applied before the antibiotic challenge (8).

The high morbidity and mortality associated with GAS-NF antibiotic treatment failure are likely multifactorial. Poor penetration of antibiotics into the infected necrotic tissue and biofilm-like structures (11, 22) as well as GAS intracellular localization (23, 24) are probable causes. Now we also show antibiotic tolerance, adding another possible explanation for antibiotic treatment failure and why surgical debridement remains essential in treating GAS-NF. Conditions encountered in the host, such as acidic pH or nutrient stress found in necrotic tissue, intracellularly in lysosomes or biofilms, and the presence of antibiotics used for treatment, induce or select for a GAS sub-population resilient to antibiotic treatment (2, 10). All the patients included in this study had been treated with high antibiotic doses before surgery. The bacteria were therefore potentially exposed to antibiotics at the time of surgical debridement but remained viable upon plating. Histology showed the presence of dense bacterial aggregates forming biofilm-like structures (11, 22) and intracellular bacteria were detected in all samples. Bacteria freshly recovered from patient tissue showed different degrees of delayed colony appearance time, resulting in heterogeneous colony sizes at an endpoint.

The laboratory strains GAS WT (12) and its isogenic *covS* mutant GAS AP, characterized by increased expression of several GAS virulence factors (13), were used as a benchmark for GAS behavior. No specific comparison of the clinical presentation of the patients, in relation to the presence of a mutation in *covS* in the invading strain could be carried out in this patient cohort, as age and elapsed time before medical intervention differed too greatly and the sample number (*n* = 6) was not sufficient to correlate any variable with clinical outcome. More detailed studies with larger cohorts are needed in this direction to establish whether persister formation and severity of the clinical presentation are influenced by the presence of a mutation in the CovR/S regulatory system. To investigate the extent to which the genetic background of an infecting strain contributes to its ability to form persisters, the comparison of more genetically diverse isolates is needed. Including different clinical presentations will also provide the opportunity to explore whether persisters occur in various GAS infections or are characteristic of NF.

In conclusion, we demonstrated that GAS recovered from NF tissue during surgery, displayed delayed colony appearance times both in patients and mice, indicative of the presence of persisters in NF tissue. *In vitro*, we showed that persister formation is triggered by exposing bacteria to stressors mimicking environmental conditions found in NF tissue, namely nutrient starvation and acidic pH. Shifting the treatment focus to a regimen that also targets persister cells could help contain the number of surviving bacteria (5, 25) contributing to reducing GAS-NF treatment failure.

## SUPPLEMENTAL MATERIAL

Figure S1 msphere.00634-24-s0001.pdf Phylogenetic tree.

Figure S2 msphere.00634-24-s0002.pdf CovS sequences.

Figure S3 msphere.00634-24-s0003.pdf Colony appearance time of GAS WT isolated from mouse tissue.

Figure S4 msphere.00634-24-s0004.pdf Time-kill curves.

Legends msphere.00634-24-s0005.docx Supplemental figure legends.

ASM does not own the copyrights to Supplemental Material that may be linked to, or accessed through, an article. The authors have granted ASM a non-exclusive, world-wide license to publish the Supplemental Material files. Please contact the corresponding author directly for reuse.
