# Supplementary figures and images for: Investigating group A Streptococcus antibiotic tolerance in necrotizing fasciitis
Source: mSphere. 2024 Aug 27;9(9):e00634-24. doi: 10.1128/msphere.00634-24 (PMC11423592; doi:10.1128/msphere.00634-24)

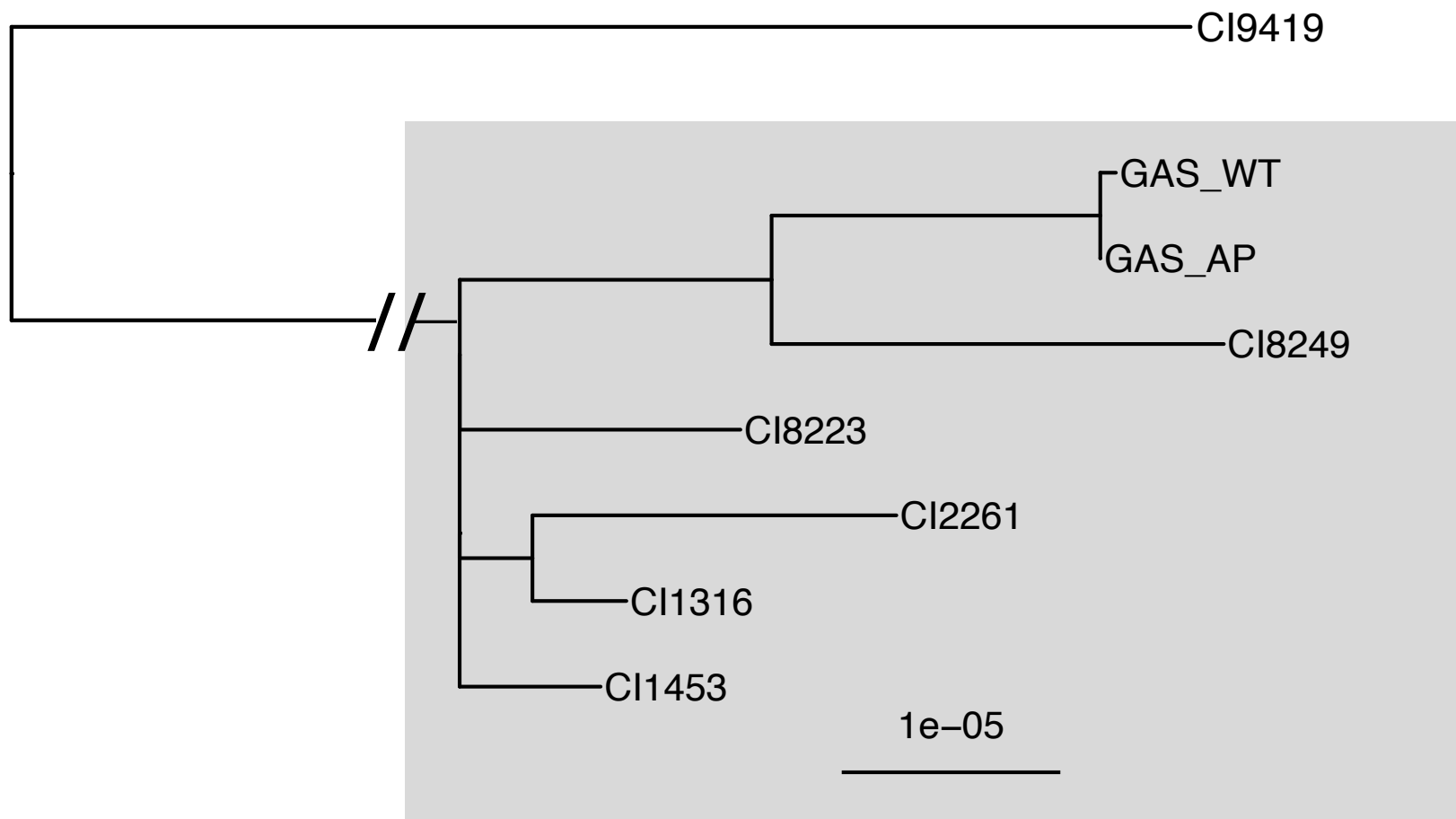

Supplement: Figure S1 — Phylogenetic tree. [file msphere.00634-24-s0001.pdf]

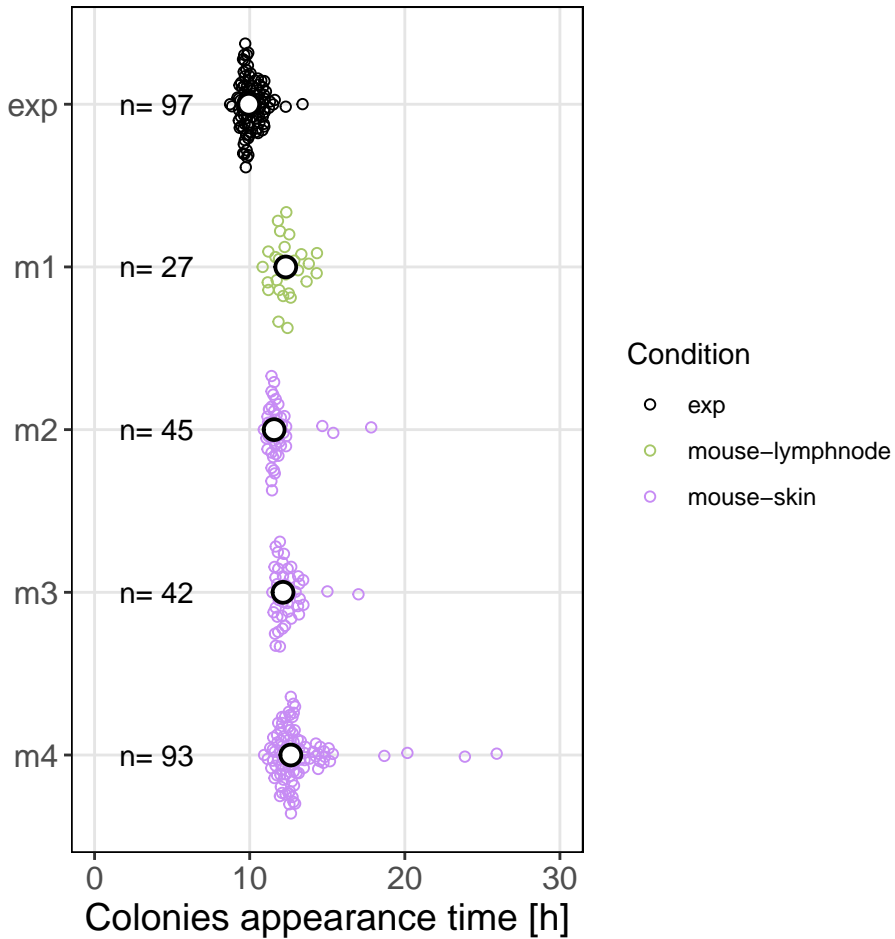

Supplement: Figure S3 — Colony appearance time of GAS WT isolated from mouse tissue. [file msphere.00634-24-s0003.pdf]

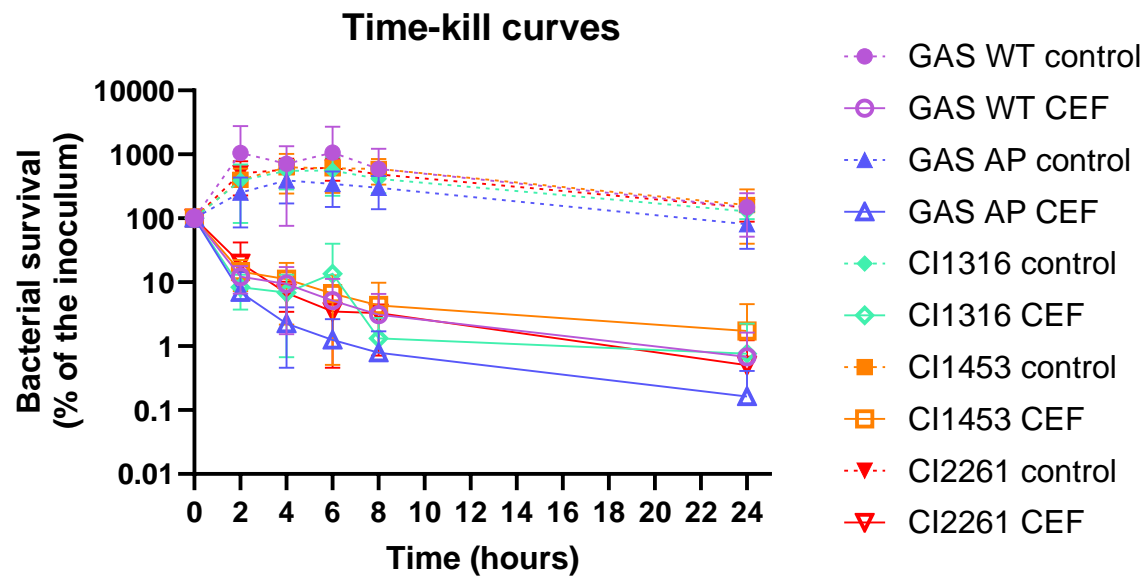

Supplement: Figure S4 — Time-kill curves. [file msphere.00634-24-s0004.pdf]
