# Supplementary material for: Investigating group A Streptococcus antibiotic tolerance in necrotizing fasciitis
Source: mSphere. 2024 Aug 27;9(9):e00634-24. doi: 10.1128/msphere.00634-24 (PMC11423592; doi:10.1128/msphere.00634-24)
Supplement: Figure S2 — CovS sequences. [file msphere.00634-24-s0002.pdf]

```
    WT MENQKQKQKKYKNSLPKRLSNIFFVLFFCIFSAFTLIAYSSTNYFLLKKEKQSVFQAVNIVRVRLSEVDSNFTLENLAEVLYKNDKTHLRIDDRKGSRVI 100
    AP MENQKQKQKKYKNSLPKRLSNIFFVLFFCIFSAFTLIAYSSTNYFLLKKEKQSVFQAVNIVRVRLSEVDSNFTLENLAEVLYKNDKTHLRIDDRKGSRVI 100
CI1453 MENQKQKQKKYKNSLPKRLSNIFFVLFFCIFSAFTLIAYSSTNYFLLKKEKQSVFQAVNIVRVRLSEVDSNFTLENLAEVLYKNDKTHLRIDDRKGSRVI 100
CI2261 MENQKQKQKKYKNSLPKRLSNIFFVLFFCIFSAFTLIAYSSTNYFLLKKEKQSVFQAVNIVRVRLSEVDSNFTLENLAEVLYKNDKTHLRIDDRKGSRVI 100
CI1316 MENQKQKQKKYKNSLPKRLSNIFFVLFFCIFSAFTLIAYSSTNYFLLKKEKQSVFQAVNIVRVRLSEVDSNFTLENLAEVLYKNDKTHLRIDDRKGSRVI 100
CI8223 MENQKQKQKKYKNSLPKRLSNIFFVLFFCIFSAFTLIAYSSTNYFLLKKEKQSVFQAVNIVRVRLSEVDSNFTLENLAEVLYKNDKTHLRIDDRKGSRAI 100
CI8249 MENQKQKQKKYKNSLPKRLSNIFFVLFFCIFSAFTLIAYSSTNYFLLKKEKQSVFQAVNIVRVRLSEVDSNFTLENLAEVLYKNDKTHLRIDDRKGSRVI 100
CI9419 MENQKQKQKKYKNSLPKRLSNIFFVLFFCIFSAFTLIAYSSTNYFLLKKEKQSVFQAVNIVRVRLSEVDSNFTLENLAEVLYKNDKTHLRIDDRKGSRVI 100

    WT RSERDITNTLDANQDIYVYNIDKQMIFTTDNEESSPGLHGPIGRVYHDHIEDQYRGFSMTQKVYSNRTGKFVGYVQVFHDLGNYYVIRARLLFWLLVVEL 200
    AP RSERDITNTLDANQDIYVYNIDKQMIFTTDNEESSPGLHGPIGRVYHDHIEDQYRGFSMTQKVYSNRTGKFVGYVQVFHDLGNYYVIRARLLFWLLVVEL 200
CI1453 RSERDITNTLDANQDIYVYNIDKQMIFTTDNEESSPGLHGPIGRVYHDHIEDQYRGFSMTQKVYSNRTGKFVGYVQVFHDLGNYYVIRARLLFWLLVVEL 200
CI2261 RSERDITNTLDANQDIYVYNIDKQMIFTTDNEESSPGLHGPIGRVYHDHIEDQYRGFSMTQKVYSNRTGKFVGYVQVFHDLGNYYVIRARLLFWLLVVEL 200
CI1316 RSERDITNTLDANQDIYVYNIDKQMIFTTDNEESSPGLHGPIGRVYHDHIEDQYRGFSMTQKVYSNRTGKFVGYVQVFHDLGNYYVIRARLLFWLLVVEL 200
CI8223 RSERDITNTLDANQDIYVYNIDKQMIFTTDNEESSPGLHGPIGRVYHDHIEDQYRGFSMTQKVYSNRTGKFVGYVQVFHDLGNYYVIRARLLFWLLVVEL 200
CI8249 RSERDITNTLDANQDIYVYNIDKQMIFTTDNEESSPGLHGPIGRVYHDHIEDQYRGFSMTQKVYSNRTGKFVGYVQVFHDLGNYYVIRARLLFWLLVVEL 200
CI9419 RSERDITNTLDANQDIYVYNIDKQMIFTTDNEESSPGLHGPIGRVYHDHIEDQYRGFSMTQKVYSNRTGKFVGYVQVFHDLGNYYVIRARLLFWLLVVEL 200

    WT FGTSLAYLIILITTRRFLKPLHNLHEVMRNISENPNNLNLRSDISSGDEIEELSVIFDNMLDKLETHTKLQSRFISDVSHELRTPVAIIKGHIGLLQRWG 300
    AP FGTSLAYLIILITTRRFLKPLHNLHEVMRNISENPNNLNLRSDISSGDEIEELSVIFDNMLDKLETHTKLQSRFISDVSHELRTPVAIIKGHNWSLTTLG 300
CI1453 FGTSLAYLIILITTRRFLKPLHNLHEVMRNISENPNNLNLRSDISSGDEIEELSVIFDNMLDKLETHTKLQSRFISDVSHELRTPVAIIKGHIGLLQRWG 300
CI2261 FGTSLAYLIILITTRRFLKPLHNLHEVMRNISENPNNLNLRSDISSGDEIEELSVIFDNMLDKLETHTKLQSRFISDVSHELRTPVAIIKGHIGLLQRWG 300
CI1316 FGTSLAYLIILITTRRFLKPLHNLHEVMRNISENPNNLNLRSDISSGDEIEELSVIFDNMLDKLETHTKLQSRFISDVSHELRTPVAIIKGHIGLLQRWG 300
CI8223 FGTSLAYLIILITTRRFLKPLHNLHEVMRNISENPNNLNLRSDISSGDEIEELSVIFDNMLDKLETHTKLQSRFISDVSHELRTPVAIIKGHIGLLQRWG 300
CI8249 FGTSLAYLIILITTRRFLKPLHNLHEVMRNISENPNNLNLRSDISSGDEIEELSVIFDNMLDKLETHTKLQSRFISDVSHELRTPVAIIKGHIGLLQRWG 300
CI9419 FGTSLAYLIILITTRRFLKPLHNLHEVMRNISENPNNLNLRSDISSGDEIEELSVIFEIC---------------------------------------- 260

    WT KDDSDILEESLTATAHEADRMAIMINDMLDMVRVQGSFEGHQNDMTVLEDSIETVVGNFRVLREDFIFTWQSENPKTIARIYKNHFEQALMILIDNAVKY 400
    AP ---------------------------------------------------------------------------------------------------- 300
CI1453 KDDSDILEESLTATAHEADRMAIMINDMLDMVRVQGSFEGHQNDMTVLEDSIETVVGNFRVLREDFIFTWQSENPKTIARIYKNHFEQALMILIDNAVKY 400
CI2261 KDDSDILEESLTATAHEADRMAIMINDMLDMVRVQGSFEGHQNDMTVLEDSIETVVGNFRVLREDFIFTWQSENPKTIARIYKNHFEQALMILIDNAVKY 400
CI1316 KDDSDILEESLTATAHEADRMAIMINDMLDMVRVQGSFEGHQNDMTVLEDSIETVVGNFRVLREDFIFTWQSENPKTIARI------------------- 381
CI8223 KDDSDILEESLTATAHEADRMAIMINDMLDMVRVQGSFEGHQNDMTVLEDSI------------------------------------------------ 352
CI8249 KDDSDILEESLTATAHEADRMAIMINDMLDMVRVQGSFEGHQNDMTVLEDSIETVVGNFRVLREDFIFTWQSENPKTIARIYKNHFEQALMILIDNAVKY 400
CI9419 ---------------------------------------------------------------------------------------------------- 260

    WT SRKEKKIAINLSVTGKQEAIVRVQDKGEGISKEDIEHIFERFYRTDKSRNRTSTQAGLGIGLSILKQIVDGYHLQMKVESELNEGSVFILHIPLAQSKES 500
    AP ---------------------------------------------------------------------------------------------------- 300
CI1453 SRKEKKIAINLSVTGKQEAIVRVQDKGEGISKEDIEHIFERFYRTDKSRNRTSTQAGLGIGLSILKQIVDGYHLQMKVESELNEGSVFILHIPLAQSKES 500
CI2261 SRKEKKIAINLSVTGKQEAIVRVQDKGEGISKEDIEHIFERFYRTDKSRNRTSTQAGLGIGLSILKQIVDGYHLQMKVESELNEGSVFILHIPLAQSKES 500
CI1316 ---------------------------------------------------------------------------------------------------- 381
CI8223 ---------------------------------------------------------------------------------------------------- 352
CI8249 SRKEKKIAINLSVTGKQEAIVRVQDKGEGISKEDIEHIFERFYRTDKSRNRTSTQAGLGIGLSILKQIVDGYHLQMKVESELNEGSVFILHIPLAQSKES 500
CI9419 ---------------------------------------------------------------------------------------------------- 260
```
