# Supplementary material for: Investigating group A Streptococcus antibiotic tolerance in necrotizing fasciitis
Source: mSphere. 2024 Aug 27;9(9):e00634-24. doi: 10.1128/msphere.00634-24 (PMC11423592; doi:10.1128/msphere.00634-24)
Supplement: Legends — Supplemental figure legends. [file msphere.00634-24-s0005.docx]

**Supplementary figure legends**

**Figure S1 – Phylogenetic tree of the GAS strains**

Maximum-likelihood phylogenetic tree based on the core alignment of 1.4 Gbp. The isolate CI9419 is distantly related to the others and serves as outgroup. To visualize distinctions among the *emm*-1 isolates, the box in grey after the cut branch represents a zoom on their clade. The scale-bar applies to the grey clade and represents a distance of ~ 14 substitutions.

**Figure S2 – CovS protein sequences alignment.**

Alignment of the CovS protein sequences. Diversion from the GAS WT (WT) sequence are marked in red. GAS AP (AP), CI1316, CI8223 and CI9419 are characterized by a truncation of the CovS C‑terminal region.

**Figure S3 – Colony appearance time of GAS WT isolated from mouse tissue**

Four mice were infected in each leg with GAS M1T1 5448 WT. The bacteria isolated from the tissue were plated, and time lapses carried out. Empty dots represent individual colonies, and the number of colonies on each plate is shown on the left (n). The interquartile range and median of each distribution are depicted with a black line and a white dot respectively. The colony appearance time of GAS WT exponential cultures (exp) were compared to the one of GAS WT directly recovered from mouse skin or lymph nodes. Bacteria recovered from mice displayed a larger median colony appearance time as compared to bacteria cultivated from exponential phase growth (i.e. on average 12.2 hours versus 9.7 hours). The data generated from exponential growth cultures (exp) are the same as the ones displayed in Fig.2A. m=mouse.

**Figure S4 – Time-kill curves**

Time-kill curves were carried out on stationary phase cultures of GAS grown overnight in THY medium and subsequently exposed to 40x MIC of ceftriaxone in THY medium. The O/N culture density was adjusted to 5x10^7^ CFUs/ml in fresh THY prior to antibiotic exposure. Bacterial viability was assessed at 2, 4, 6, 8 and 24 hours and is depicted in the graph as a percentage of the initial inoculum (5x10^7^ CFUs/ml).
